# Erythrocyte fatty acid aberrations in Amyotrophic Lateral Sclerosis: Correlation with disease duration

**DOI:** 10.5937/jomb0-40387

**Published:** 2023-10-27

**Authors:** Rajna Minić, Aleksandra Arsić, Milica Kojadinović, Aleksa Palibrk, Brizita Đorđević, Zorica Stević

**Affiliations:** 1 University of Belgrade, Institute for Medical Research, National Institute of Republic of Serbia, Belgrade; 2 University of Belgrade, School of Medicine, Clinical Center of Serbia, Neurology Clinic, Belgrade; 3 University of Belgrade, Faculty of Pharmacy, Belgrade

**Keywords:** amyotrophic lateral sclerosis, metabolism, monounsaturated fatty acids, polyunsaturated fatty acids, saturated fatty acids, amiotrofična lateralna skleroza, metabolizam, mononezasičene masne kiseline, polinezasičene masne kiseline, zasičene masne kiseline

## Abstract

**Background:**

Recent literature data highlights metabolic changes in amyotrophic lateral sclerosis (ALS). To explore possible early metabolic changes, we aimed to analyse the fatty acids (FA) composition of erythrocytes in newly diagnosed als patients and to see whether fatty acid levels correlate with the ALSFRS-R score or disease duration.

**Methods:**

The severity of motor function involvement was assessed by the ALSFRS-R scale at the initial evaluation. The fatty acid profile of erythrocyte membranes was analysed by gas-liquid chromatography. The study comprised 26 clinically diagnosed als patients, with mean ALSFRS-R 38±8. The control group included 26 healthy volunteers.

## Abstract (sr)

**Uvod:**

Dostupni literaturni podaci ističu značaj metaboličkih promena kod amiotrofične lateralne skleroze (ALS). Sa ciljem da istražimo moguće rane metaboličke promene analizirali smo sastav masnih kiselina (MK) eritrocita kod novodijagnostikovanih als pacijenata da bi videli da li nivoi masnih kiselina koreliraju sa ALSFRS-R ocenom ili trajanjem bolesti.

**Metode:**

Stepen oštećenja motoričke funkcije procenjivan je ALSFRS-R skalom pri početnoj proceni. Studija je obuhvatila 26 klinički dijagnostikovanih als pacijenata, srednja vrednost ALSFRS-R bila je 38±8. Kontrolnu grupu činilo je 26 zdravih dobrovoljaca. Profil masnih kiselina membrana eritrocita je analiziran gasno-tečnom hromatografijom.

**Rezultati:**

Značajno viši nivoi palmitinske kiseline i ukupnih zasićenih MK nađeni su kod als pacijenata. Ukupne mononezasičene MK, palmitoleinska, vakcenska i oleinska kiselina takođe su bile značajno povečane kod als pacijenata. Nivoi eikozapentaenske kiseline, dokozapentaenske kiseline, ukupne polinezasičene MK (PUFA) i n-6 PUFA bili su značajno niži kod pacijenata sa als. Pored toga, alinolenska kiselina, prekursor porodice n-3 PUFA, uopšte nije detektovana kod als pacijenata. Nismo pronašli značajnu korelaciju između ALSFRS-R ocene i nivoa pojedinačnih analiziranih MK. Umerena negativna korelacija je pronađena između trajanja bolesti i nivoa DHA, a nađena je pozitivna korelacija trajanja bolesti sa nivoom MUFA.

**Zaključak:**

Prikazani eksperimentalni dokazi mogu doprineti oblikovanju korisne nutritivne intervencije.

## Introduction

Amyotrophic lateral sclerosis (ALS) is the most frequent fatal neurodegenerative disorder in people aged 50–70. In most cases, it has a short duration from diagnosis until death. The pathogenic processes in ALS are complex and incompletely understood, and numerous gene mutations are involved. The selective degeneration of motor neurons results in progressive paralysis of voluntary muscles, with the cause of death generally being respiratory failure [1] [2] [3]. The primary reason for motor neurons suddenly dying is still elusive and therapeutic interventions that could reverse the conditions are lacking.

Hypermetabolism was noted in two-thirds of ALS patients, and there is also a high incidence of glucose intolerance and dyslipidaemia. Due to hypermetabolism, weight loss occurs with a decline in nutritional status [4] [5] which is linked with a worse disease prognosis [6]. Studies suggest that lipids as energy sources could benefit ALS, although no official recommendations exist. In many studies, high levels of total cholesterol and LDL-cholesterol are found in the ALS population [7] [8]. Higher triglyceride levels were also found in women with ALS [9], and hypertriglyceridemia was associated with better functional status and prolonged survival [10]. In our earlier investigation, dyslipidemia was found in over 50% of ALS patients, unrelated to significantly longer survival [7]. Besides lipid profile alterations, lipid metabolism alterations were previously reported in neurons and skeletal muscle in rodent ALS models [11] [12] [13].

Efforts to thoroughly document changes in lipid metabolism and to detect biomarkers for the disease are being made, as well as to provide recommendations for future nutritional interventions in ALS patients. The decision on possible nutritional interventions in ALS patients relies on clinical studies, but the analysis of blood fatty acid (FA) composition in patients represents a useful tool, especially the analysis of erythrocyte membrane FA composition. The FA composition of erythrocyte membranes reflects the dietary intake and endogenous lipid metabolism for a relatively long period. The goals of the present study were to analyse the lipid profile and fatty acid composition of erythrocyte membranes in newly diagnosed ALS patients and to investigate if any correlation with the ALSFRS-R score or disease duration exists.

## Materials and methods

### Study subjects

This study included 26 ALS patients (9 females, 17 males; mean age 63±9) clinically diagnosed at the Clinic of Neurology, Clinical Center of Serbia. All patients fulfilled diagnostic criteria for definite or probable ALS according to the El Escorial revised criteria – R. There were 18 patients with spinal and 8 with a bulbar presentation at the onset of the disease. Exclusion criteria were patients who took lipid-lowering medication, those supplemented with antioxidants, fish oil, or other supplements which influence lipid metabolism for at least 6 weeks before the study, and those on restrictive diets.

The severity of motor function involvement was assessed at the initial evaluation by the ALSFRS-R scale (R) [14]. Individual item scores are summed, and the final score lies between 0=worst and 48=best.

Disease duration was measured from initial plasma sampling until death or for patients still alive until the present moment.

The study was conducted following the Declaration of Helsinki and principles of Good Clinical Practice and with approval from the local Ethical Committee (School of Medicine, University of Belgrade, Serbia). The control group included 26 healthy sex-matched volunteers. All subjects gave written participation consent.

### Biochemical analyses

Blood samples were taken after an overnight fast. Lipid status and glucose level are measured immediately after blood collection, using the automated clinical analyser Dade Behring Dimension RxL (Siemens AG, Munich, Germany).

### Fatty Acid Extraction and Analysis

Plasma was collected into 3 mL EDTA tubes. Thrombocytes were removed by centrifugation at 180 x g. Erythrocytes were washed three times with equal volumes of physiological solution and pelleted by centrifugation 1800xg. Lipids from erythrocyte membranes were extracted with a mixture of chloroform/methanol (2:1, v/v) according to the previously described method [15]. Fatty acid methyl esters (FAME) obtained by transesterification with 3 mol/L HCl in methanol were analysed by gas-liquid chromatography using a Shimadzu chromatograph GC 2014 (Kyoto, Japan) equipped with a flame ionisation detector. The column was Rtx 2330 (60 m × 0.25 mmID, with a film thickness of 0.2 μm, RESTEK, Bellefonte, PA, USA). Individual FAME was identified by comparing peak retention times with retention times of standardised mixtures (PUFA-2 and/or 37 FAMEs mix, Supelco, Bellefonte, PA, USA).

The following fatty acids were analyzed: 16:0 palmitic acid, 18:0 stearic acid, 16:1n-7 palmitoleicacid, 18:1n-7 vaccenic acid, 18:1n-9 oleic acid, 18:2n-6 linoleic acid, 18:3n-6 g-linolenic acid, 20:3n-6 dihomo-gamma-linolenic acid, 20:4n-6 arachidonic acid (AA), 22:4n-6 docosatetraenoic acid, 18:3n-3 a-linoleic acid (ALA), 20:5n-3 eicosapentaenoic acid (EPA), 22:5n-3 docosapentaenoic acid (DPA), 22:6n-3 docosahexaenoic acid (DHA). The content of individual FA was expressed as a percentage of the total fatty acids identified.

### Desaturase and elongase indices

To estimate the activities of certain enzymes involved in FA biosynthesis, product-to-precursor ratios in erythrocytes were used, in particular: 16:1n-7/16:0 ratio for Stearoyl-CoA desaturase (SCD-16), 18:1n-9/18:0 ratio for Stearoyl-CoA desaturase (SCD-18) activity, 18:3n-6/18:2n-6 ratio for delta-6-desaturase (D6-desaturase) activity, 20:4n-6/20:3n-6 ratio for delta-5-desaturase (D5-desaturase) activity, and 18:0/16:0 for elongase activity.

### Inflammation status and peroxidability index

The inflammation status (IS) can be inferred from arachidonate AA to eicosapentaenoic EPA ratio, IS = 20:4 (n-6)/ 20:5 (n-3) [16] [17].

Peroxydability index (PI) was calculated using the following formula: PI = (% monoenoic FA×0.025) (% dienoic FA×1) (% trienoic FA×2) (% tetraenoic FA×4) (% pentaenoic FA×6) (% hexaenoic FA×8) [18].

### Statistical analysis

Statistical analysis was performed with GraphPad Prism software. The normality of distribution was evaluated with the Shapiro-Wilk normality test, the Mann-Whitney test was used for group comparison, and p<0.05 was considered statistically significant. Pearson correlation was used to measure the linear correlation between two variables.

## Results

Patient characteristics are provided in Table 1.

**Table 1:** Characteristic of study population and biochemical parameters in ALS and control groups. Data are presented as median (IQR). Probability – p; ^*^ - p<0.05; ^***^ - p<0.0005.

Parameters	ALS group (N=26)	Control group (N=26)	Probability
Age	64 (56.5–69.75)	55.5 (46.75–60.0)	0.0003^***^
Gender - male	17	17	/
BMI	25.5 (24.05–26.99)	23.87 (21.3–27.55)	0.2906
Glucose (mmol/L)	4.95 (4.6–5.5)	4.32 (4.04–4.68)	0.0001^***^
Triglycerides (mmol/L)	1.65 (1.18–2.20)	0.82 (0.61–1.22)	0.0001^***^
Cholesterol (mmol/L)	5.10 (4.53–5.75)	4.63 (3.71–5.07)	0.0082^*^
HDL-cholesterol (mmol/L)	1.41 (1.19–1.83)	1.51 (1.40–1.68)	0.2926
LDL-cholesterol (mmol/L)	3.00 (2.58–3.40)	2.64 (1.81–2.93)	0.0138^*^
LDL/HDL	2.14 (1.56–2.5)	1.64 (1.20–1.96)	0.0131^*^
ALSFRS-R score	38±8	48	/

In comparison to the reference values obtained from the publication of the National Cholesterol Program (NCEP) Adult Treatment Panel III [19], the ALS group had cholesterol levels at the upper limit of the reference value (5.1 mmol/L), and increased triglycerides (reference value 1.7 mmol/L). Also, 11.5% of patients had hyperglycemia, 46.1% had hypertriglyceridemia, and 50.0% had hypercholesterolemia. In this cohort, 36.36% of ALS patients had a lower level of HDL-cholesterol (Table 1).

*FA composition of erythrocytes is shown in* Table 2. We found significantly higher palmitic, palmitoleic, oleic and vaccenic acid levels in the ALS group. Also, ALA, the precursor of the n-3 PUFA family, was not detected in ALS patients. Further, its products EPA and DPA were lower in all ALS patients than in controls. Significantly higher inflammatory process estimation (AA/EPA) was found in ALS patients, and a significantly lower level of the peroxidability index (p=0.0078).

**Table 2:** Erythrocytes phospholipids fatty acid composition. Data are presented as median (IQR). EPA+DHA = omega-3 indeks, AA/EPA= inflammation status, PI – peroxydability index = (% monoenoic FA×0.025) (% dienoic FA×1) (% trienoic FA×2) (% tetraenoic FA×4) (% pentaenoic FA×6) (% hexaenoic FA×8). Probability – p; ^*^–p<0.05; ^**^–p<0.005; ^***^–p<0.0005.

Fatty acids (%)	ALS group	Control group	p
	(n=26)	(n=26)	
16:00	24.97 (24.16–26.37)	23.65 (23.25–24.17)	0.0002^***^
18:00	18.82 (17.57–20.93)	18.33 (17.56–18.69)	0.0859
SFA	43.17 (41.95–46.84)	41.96 (41.24–42.25)	0.0019^**^
16:1 n-7	0.355 (0.19–0.45)	0.22 (0.14–0.30)	0.0294^*^
18:1 n-7	1.26 (1.01–1.35)	1.06 (0.98–1.19)	0.0044^*^
18:1 n-9	13.07 (12.33–13.46)	12.33 (11.53–12.61)	0.0107^*^
MUFA	14.68 (14.06–15.06)	13.59 (13.05–13.94)	0.0006^**^
n-6 PUFA	36.83 (32.04–37.96)	38.3 (37.24–38.93)	0.0016^**^
18:2 n-6	10.29 (9.51–11.03)	11.54 (10.62–12.49)	0.0068^*^
18:3 n-6	0.435 (0.36–0.53)	0.425 (0.39–0.52)	0.8116
20:3 n-6	3.20 (2.7–3.63)	3.35 (2.51–4.08)	0.8908
20:4 n-6	18.04 (16.09–20.10)	19.19 (17.62–20.12)	0.1014
22:4 n-6	3.78 (3.24–4.16)	4.04 (3.17–4.55)	0.2201
n-3 PUFA	5.57 (4.81–6.47)	6.11 (5.21–7.30)	0.0386^*^
18:3 n-3	0	0.07 (0.05–0.12)	/
20:5 n-3	0.23 (0.17–0.30)	0.34 (0.22–0.47)	0.0059^*^
22:5 n-3	1.43 (1.16–1.58)	1.65 (1.53–1.93)	0.0014^**^
22:6 n-3	3.85 (3.06–4.49)	3.98 (3.42–4.70)	0.2926
Total PUFA	42.49 (38.53–43.83)	44.14 (43.57–45.32)	0.0002^***^
EPA+DHA	4.05 (3.25–4.78)	4.32 (3.61–5.32)	0.1816
AA/EPA	78.04 (61.44–93.76)	57.88 (39.38–87.85)	0.0301^*^
PI	150.7 (131.8–154.2)	156.4 (150–162)	0.0078^*^

Total SFA and MUFA were increased (p=0.019 and p=0.0006, respectively), and total PUFA and n-6 PUFA were decreased in ALS patients (p=0.0002, p=0.016, respectively) (Table 2).

Estimated enzymes elongase and desaturase activities were not different between the examined groups (Table 3).

**Table 3:** Estimated activity of desaturase and elongase in erythrocytes in ALS patients and controls. Data are presented as median (IQR). Probability–p.

Desaturase<br>and elongase	ALS group<br>(n=26)	Control group<br>(n=26)	p
SCD-16	0.01 (0.01–0.02)	0.01 (0.01–0.01)	0.1976
SCD-18	0.67 (0.63–0.71)	0.68 (0.63–0.71)	0.9561
Δ6 desaturase	0.04 (0.03–0.05)	0.04 (0.03–0.05)	0.1837
Δ5 desaturase	5.65 (4.82–7.02)	5.71 (4.64–7.59)	0.4530
elongase	0.76 (0.73–0.80)	0.78 (0.75–0.80)	0.4738

When we classified patients into two groups as being normolipidemic or dyslipidemic and analysed their fatty acid profile, we mainly confirmed the differences in total MUFA and PUFA found in combined groups. Hence, we found differences between the two ALS groups, such as the levels of palmitoleic acid and AA/EPA were significantly higher, while the level of linoleic acid was significantly lower only in normolipidemic ALS patients (Table 4). On the other hand, only the dyslipidemic group had significantly higher oleic acid and significantly lower PI than the control group. We need to stress that the dyslipidemic group was 60% larger than the normolipidemic group.

**Table 4:** Fatty acid profiles in normolipidemic and dislipidemic ALS patients. Data are presented as median (IQR). EPA+DHA = omega-3 indeks, AA/EPA= inflammation status, PI – peroxydability index = (% monoenoic FA×0.025)＋ (% dienoic FA×1)＋(% trienoic FA×2)＋(% tetraenoic FA×4)＋(% pentaenoic FA×6)＋(% hexaenoic FA×8). Significance was calculate in comparison to the control group. Probability – p; ^*^–p<0.05; ^**^–p<0.005.

Fatty acids<br>(%)	Normolipidemic ALS group<br>n=10	p	Dyslipidemic ALS group<br>n=16	p	Control group<br>n=26
16:00	25.09 (24.44–26.81)	0.012^*^	24.97 (23.78–26.71)	0.003^**^	23.65 (23.25–24.17)
18:00	18.9 (17.04–21.18)	0.3869	18.74 (18.01–21.36)	0.0874	18.33 (17.56–18.69)
SFA	43.01 (41.43–47.53)	0.0745	43.17 (42.12–48.00)	0.0021^**^	41.96 (41.24–42.25)
16:1 n-7	0.41 (0.31–0.73)	0.0155^*^	0.26 (0.19–0.39)	0.1951	0.22 (0.14–0.30)
18:1 n-7	1.26 (0.97–1.54)	0.046^*^	1.24 (1.04–1.33)	0.0107^*^	1.06 (0.98–1.19)
18:1 n-9	12.8 (12.08–13.56)	0.0934	13.26(12.43–13.52)	0.0178^*^	12.33 (11.53–12.61)
MUFA	14.38 (14.01–15.57)	0.005^*^	14.81 (14.00–15.02)	0.0052^*^	13.59 (13.05–13.94)
18:2 n-6	9.935 (8.72–11.11)	0.0105^*^	10.60 (10.04–11.38)	0.0521	11.54 (10.62–12.49)
18:3 n-6	0.415 (0.36–0.49)	0.697	0.47 (0.38–0.53)	0.5252	0.43 (0.39–0.52)
20:3 n-6	3.15 (2.74–3.57)	0.6976	3.29 (2.62–3.68)	0.9381	3.35 (2.51–4.08)
20:4 n-6	18.55 (16.03–20.16)	0.5022	17.91 (15.69–19.97)	0.0698	19.19 (17.62–20.12)
22:4 n-6	3.835 (3.11–4.19)	0.4689	3.63 (3.28–4.11)	0.2334	4.04 (3.17–4.55)
n-6 PUFA	37.1 (31.34–38.25)	0.0326^*^	36.34 (32.14–37.77)	0.0039^**^	38.30 (37.24–38.93)
18:3 n-3	0	/	0	/	0.07 (0.05–0.12)
20:5 n-3	0.19 (0.11–0.27)	0.0099^*^	0.25 (0.18–0.30)	0.046^*^	0.34 (0.22–0.47)
22:5 n-3	1.42 (1.08–1.62)	0.0121^*^	1.50 (1.21–1.60)	0.0073^*^	1.65 (1.53–1.93)
22:6 n-3	4.00 (2.95–4.44)	0.5022	3.73 (3.10–4.58)	0.325	3.98 (3.42–4.70)
n-3 PUFA	5.52 (4.55–6.62)	0.1334	5.66 (4.85–6.37)	0.0698	6.11 (5.21–7.30)
total PUFA	42.57 (37.41–44.18)	0.0077^*^	42.35 (37.15–43.59)	0.0007^**^	44.14 (43.57–45.32)
EPA+DHA	4.27 (3.11–4.71)	0.3869	3.99 (3.29–4.87)	0.2138	4.33(3.61–5.32)
AA/EPA	96.8 (66.38–121.80)	0.0224^*^	68.90 (61.08–85.32)	0.1659	57.82 (39.25–88.12)
PI	151.3 (139.9–154.3)	0.0804	149.7 (125.5–155.7)	0.0134^*^	156.4 (150.0–162.0)

**Figure 1:**
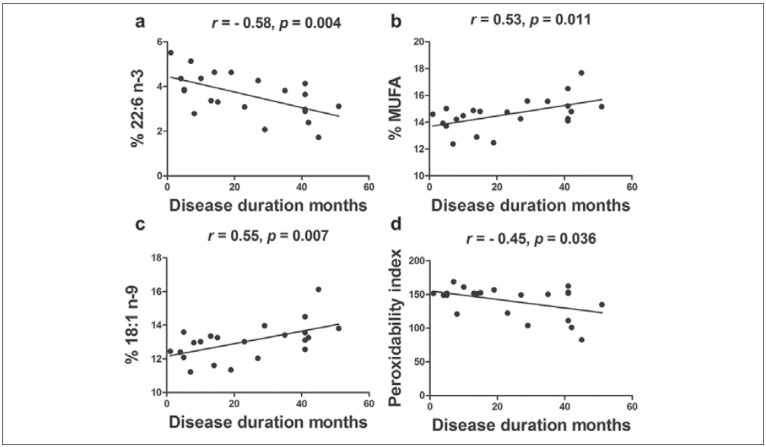
Correlation of disease duration with A) DHA levels, B) MUFA levels, C) oleic acid levels and D) Peroxidability index. Disease duration was measured from initial plasma sampling until death or until the present moment. r – Pearson’s correlation coefficient, p – probability, calculated in GraphPad Prism software. 22:6 n- 3 – DHA; MUFA – monounsaturated fatty acids; 18:1 n-9 oleic acid; peroxidability index calculated according to the formula: peroxydability index = (% monoenoic FA×0.025) (% dienoic FA×1) (% trienoic FA×2) (% tetraenoic FA×4) (% pentaenoic FA×6) (% hexaenoic FA×8).

We next wanted to determine if a correlation existed between the levels of individual parameters measured and the ALSFRS-R. We found no significant correlation between the ALSFRS-R with the abundance of individual fatty acids. Non-significant correlation (r=0.4, p=0.057) was found between the ALSFRS-R score at sampling and disease duration. Disease duration showed a moderate negative correlation with the DHA level (r=-0.58, p=0.004) (Figure 1A), n-3 (r=-0.50, p=0.017), EPA+DHA (r=-0.55, p=0.008) and PI (Figure 1D) and moderate positive correlation with the level of MUFA (Figure 1B), oleic acid (Figure 1C) and palmitic acid (r=0.43, p=0.044). No significant correlation of disease duration was found with glucose, cholesterol, HDL, LDL or LDL/HDL values.

## Discussion

In this study, lipid and erythrocyte fatty acids profiles of ALS patients at the time of diagnosis were analysed to see if there are differences compared to healthy subjects and if any correlation with the ALSFRS-R score and disease duration exists. To our knowledge, this is the first study that analysed erythrocyte fatty acid content in ALS, as previous studies analysed plasma levels or total blood clot lipid content.

We have detected hypercholesterolemia and/or hypertriglyceridemia in 61.5% of ALS patients. These results agree, in general, with the results of other authors suggesting that hyperlipidemia is a typical feature of ALS [20]. Moreover, literature data points out that the observed hyperlipidemia in ALS patients is a prognostic factor for survival [20]. We found no difference in disease duration in this patient cohort concerning cholesterol level. Also, the elevated content of total cholesterol and triglycerides was confirmed in ALS patients and associated with better prognosis in several studies analysing patient cohorts from different countries and continents [20] [21] [22] or with slower disease progression in an Italian cohort [23]. Contrary, Yang et al. [24] showed that the parameters of lipid metabolism are significantly lower in men with ALS compared to controls. Nonetheless, there is evidence that significant alterations in the advanced stages of ALS occur in lipid metabolism [25] [26].

The fatty acid composition of erythrocyte membranes reflects the type of dietary intake of FA andtheir endogenous metabolism. The obtained results suggest that ALS patients had altered erythrocyte FA profiles. The high level of individual and total SFA and MUFA are consistent with the results of other authors, who also found that total and individual MUFA are higher in the blood cells of ALS patients than in controls. Moreover, Henriques et al. [27] suggested that the ratio of 16:1/16:0 in blood cells can predict the life expectancy of ALS patients in a way that patients with a higher 16:1/16:0 ratio have lower lipid peroxidability and extended survival; disease in such patients progresses slower, within the observation period (six months). In the present study, we showed an increased level of both 16:0 and 16:1n-7 in ALS patients, and the ratio of 16:1/16:0 did not differ between the groups. We obtained a moderate positive correlation of 16:0 (r = 0.43) with disease duration.

While the level of stearic and palmitic acids in erythrocytes depends on both endogenous synthesis and dietary intake, levels of n-7 and n-9 unsaturated fatty acids mainly depend on synthesis from endogenous precursors [28] [29]. Thus, high levels of palmitoleic and vaccenic acid, which we observed, probably indicate their increased endogenous synthesis. A higher level of oleic acid in ALS patients is more likely the result of increased synthesis rather than of high dietary intake because, in general, in the Serbian population, the intake of oleic acid is very low [30].

Higher levels of individual and total SFA are closely linked with cardiovascular diseases, but, as was nicely summarised by Area-Gomez et al. [31], it seems that the metabolic changes which occur in ALS and which are usually associated with higher risk for cardiovascular disorders, seem to be protective in ALS.

Interestingly, we did not detect ALA at all in ALS patients. These results contradict another study [27], which noted higher levels of ALA in ALS patients than in controls. But, in a recent study which analysed the prediagnostic plasma levels of fatty acids, a significant inverse association between the level of ALA and the development of ALS was found [32], which follows the results presented here. ALA is an essential FA which must be ingested with food and then converted to its products EPA, DPA and DHA [33]. However, although the conversion of ALA to its long-chain derivatives influences the reduction of its levels, so do β-oxidation and carbon recycling [34]. Thus, the reason for not detecting ALA in erythrocytes in this study may be due to low intake, fast β-oxidation, or conversion to its products. However, lower levels of EPA and DPA indicate that zero levels of ALA have resulted from low intake of this FA or some other mechanism rather than its higher conversion. Also, the omega-3 index (EPA+DHA content) is considered a good biomarkerof omega-3 fatty acid intake [35]. In this study, a lower level of EPA+DHA in ALS patients than in controls indicates that we have rapid β-oxidation of ALA, but these differences were not significant.

Also, we found lower levels of n-6 PUFA, particularly linoleic acid, in ALS patients. Lipid mediators derived from n-6 PUFA can express both pro- and anti-inflammatory activities, so altered n-6 PUFAmetabolism and lower level of PUFA may be linked to the pathogenesis of many disorders linked with inflammation [36]. The AA/EPA ratio is considered a diagnostic parameter for measuring the inflammation status or the activity of pro-inflammatory eicosanoids and cytokines [17]. In our study, the level of AA/EPA was significantly higher in ALS patients, in accordance with literature data showing subclinical inflammation in ALS patients [37].

AA and DHA are the most common FAs in brain tissues. AA is involved in many essential functions in the central nervous system, including neurotransmission [38] [39]. Nevertheless, it is unclear how well erythrocyte PUFA levels correlate with PUFA brain levels. Namely, Carver et al. reported an inverse association of DHA and AA levels in erythrocytes and brain tissue [40]. Apart from that, there is evidence that the highly peroxidisable DHA and n-3 PUFA decrease in abundance in the spinal cord samples in ALS patients while the frontal cortex increases [41]. Thus, while the brain cortex could produce anti-inflammatory docosatrienes [42] derived from n-3 PUFA, this process would be impaired in the spinal cord neurons due to decreased DHA availability [41].

Finally, we checked whether there is an initial difference in FA profile between two subgroups (hyperlipidemic and normolipidemic) of patients with ALS because there is evidence that erythrocyte PUFA concentrations are associated with very low-density lipoprotein and triglyceride concentrations in the circulation [43]. However, our results showed no difference in PUFA levels among subgroups compared with controls. These results indicate that FA content is changed in ALS patients regardless of whether lipid parameters have changed, although a larger patient cohort is needed for a more detailed analysis.

So far, there have been contradictory findings regarding PUFA supplementation; for instance, in the experimental SOD1 mouse model, supplementation with EPA alone resulted in faster disease progression and death [44], while supplementation with n-6 PUFA fatty acids also in a mouse model had positive effects [45]. The authors concluded that the ratio of PUFA is key for supplementation to be beneficial.

Our results suggest that ALS patients have lower levels of both n-3 and n-6 PUFA. It is important to stress that the changes detected here in ALS patients are different to changes occurring with ageing, as somewhat opposite changes come with ageing, such as the increase in n-3 fatty acids up to the age of 70 [46]. Here we did not detect a significant correlation of the ALSFRS-R score with disease duration or with the abundance of individual fatty acids, which could be the consequence of a limited number of patients or the fact that the majority of patients were selected at disease onset, which is why most of the ALSFRS-R scores were rather high. More importantly, we detected a negative correlation of disease duration with n-3 PUFA content, DHA in particular, and a positive correlation with MUFA content, oleic acid in particular, which corroborates the findings that, unlike other age-related diseases, the intake of n-3 fatty acids might not be beneficial in ALS.

## Dodatak

### Acknowledgements

We are thankful to the patients for participating in the study. Funding was provided by the Ministry of Science, Technological Development, and Innovation of the Republic of Serbia (No. 451-03- 47/2023-01/200015).

### Conflict of interest statement

All the authors declare that they have no conflict of interest in this work.

### List of abbreviations

AA, arachidonic acid; 

ALA, a-linolenic acid; 

ALS, Amyotrophic Lateral Sclerosis; 

ALSFRS-R scale, Revised Amyotrophic Lateral Sclerosis Functional Rating Scale; 

DHA, docosahexaenoic acid; DPA, docosapentaenoic acid; EPA, eicosapentaenoic acid; 

FA, fatty acid; 

FAME, Fatty acid methyl esters; 

HDL, high-density lipoprotein; 

IS, inflammation status; 

LDL, low-density lipoprotein; 

MUFA, monounsaturated fatty acid; 

PI, Peroxydability index; 

PUFA, polyunsaturated fatty acid; 

SCD, Stearoyl-CoA desaturase; 

SFA, saturated fatty acid; 

SOD1, superoxide dismutase.
